# Injection Molding of Coir Coconut Fiber Reinforced Polyolefin Blends: Mechanical, Viscoelastic, Thermal Behavior and Three-Dimensional Microscopy Study

**DOI:** 10.3390/polym12071507

**Published:** 2020-07-07

**Authors:** Miguel A. Hidalgo-Salazar, Juan P. Correa-Aguirre, Serafín García-Navarro, Luis Roca-Blay

**Affiliations:** 1 Research Group for Manufacturing Technologies GITEM, Universidad Autónoma de Occidente, Cali 760030, Colombia; jpcorrea@uao.edu.co; 2 AIMPLAS, Gustave Eiffel 4 (València Parc Tecnològic), 46980 Paterna, Spain; sgarcia@aimplas.es (S.G.-N.); lroca@aimplas.es (L.R.-B.)

**Keywords:** biocomposites, mechanical properties, thermal properties, natural fibers, injection molding

## Abstract

In this study, the properties of a polyolefin blend matrix (PP-HDPE) were evaluated and modified through the addition of raw coir coconut fibers-(CCF). PP-HDPE-CCF biocomposites were prepared using melt blending processes with CCF loadings up to 30% (*w*/*w*). CCF addition generates an increase of the tensile and flexural modulus up to 78% and 99% compared to PP-HDPE blend. This stiffening effect is caused by a decrease in the polymeric chain mobility due to CCF, the higher mechanical properties of the CCF compared to the polymeric matrix and could be an advantage for some biocomposites applications. Thermal characterizations show that CCF incorporation increases the PP-HDPE thermal stability up to 63 °C, slightly affecting the melting behavior of the PP and HDPE matrix. DMA analysis shows that CCF improves the PP-HDPE blend capacity to absorb higher external loads while exhibiting elastic behavior maintaining its characteristics at higher temperatures. Also, the three-dimensional microscopy study showed that CCF particles enhance the dimensional stability of the PP-HDPE matrix and decrease manufacturing defects as shrinkage in injected specimens. This research opens a feasible opportunity for considering PP-HDPE-CCF biocomposites as alternative materials for the design and manufacturing of sustainable products by injection molding.

## 1. Introduction

A polymeric blend is a material formed from the physical combination of at least two polymers. These materials are used in various technological applications due to the possibility of modifying several properties considering the main characteristics of the polymers in the blend and their mixing ratio. Some studies estimated that currently, a large quantity of the polymers in the global market are sold as polymeric blends [1]. In the research context, these materials have been studied by several authors in the last years [2,3,4]. According to these studies, polymeric blends can be classified as immiscible (heterogeneous), compatible, or miscible (homogeneous), been most of these materials (around 90%) immiscible or multiphase systems with partial miscibility [1,3,4]. 

According to a Colombian non-profit organization that represents the companies related to chemical sector including plastics, rubber, paints, inks (coatings), and fibers, ACOPLASTICOS, the Colombian production of plastic resins was about 1.34 million tons in 2017 and polyolefins (low density polyethylene—LDPE, high density polyethylene and polypropylene—PP) represent around 42% of this production capacity [5]. Also, those polymers represent most of the post-consumer plastic wastes and the separation into the individual polymer and complete sorting during mechanical recycling processes are expensive and sometimes impossible. However, valorization of these polyolefins is possible because they can be easily recycled by converting them into good performance polymer blends [6,7]. 

PP-PE blends have been studied for several researchers in the last years. Some of these studies show the limited miscibility of PP-LDPE blends [8]. However, most of the revised literature shows that PP and PE are immiscible, resulting in phase separation during melt blending, low adhesion between the constituents’ phases, and poor mechanical properties [9,10,11]. Nonetheless, due to their availability, recyclability, sustainable character, and low-cost, PP-PE blends could become strategic materials for several industry applications facing a circular plastics economy [7,12,13,14]. 

The reinforcement of polymeric matrices with natural fibers as coir coconut fiber, hemp, sisal, pineapple, sugarcane bagasse, fique, wood flour and their combinations has been studied during the last years [15,16,17,18,19,20,21,22]. These materials are known as natural fiber reinforced polymer composites (NFRPCs) or biocomposites and have the potential to be used in several applications as automobile parts, construction, and furniture due to the lower cost of natural fibers in comparison with traditional fibers and the enhancement of the polymeric matrices properties induced by natural fibers incorporation [22,23]. Also, biocomposites provide some advantages such as reduction overweight, less dependence on oil resources, lower costs and CO_2_ emissions, recycling, among others [24,25,26]. 

The coconut fruit constituents are the white meat (28% wt), which is protected by the shell (12% wt) and the coir (35% wt). Also, the raw coconut husk is formed by coir fibers (30% wt) and a cork-like material called pith (70% wt). Coir Coconut Fibers (CCF) main constituents are cellulose (42% wt), hemicellulose (0.25% wt), lignin (47% wt), ashes (2% wt), pectin (3% wt) and about 5% wt of moisture [17]. According to Alvarado [27], estimated coconut production in Colombia was around 139,000 metric tons, which generates at least 50,000 metric tons of coconut husks mainly used in the hydroponic industry, soil stabilization, compost, and fuel. 

Several studies exploring the characterization of thermoplastic polymers-CCF biocomposites have been reported. The term biocomposite is often used to name polymeric reinforced composites, where the reinforcing phase and/or the matrix are derived from materials of biological origin [28,29]. According to the reviewed literature, several studies have reported the formulation and characterization of biocomposites, which have a status of renewable and sustainable materials since they are composed of natural fibers embedded in non-degradable and biodegradable polymeric matrices [30,31,32,33]. 

Mir et al. [33] studied the incorporation of CCF (up to 20% wt) in a PP matrix using thermocompression. The CCF were chemically treated with chromium sulfate and sodium bicarbonate in hydrochloric acid media to improve the compatibility between fibers and PP. Their results show that the tensile properties of PP-CCF biocomposites change with the fiber load. For the biocomposite PP-CCF 10% wt, the tensile strength increases 11% as compared with PP, whereas for PP-CCF 20% wt, the tensile strength drops to values lower than those of PP. 

Haque et al. explored the effect of fiber content (up to 30% wt) and chemical modification of the fibers with sodium hydroxide and a benzene diazonium salt on the mechanical properties of biocomposites based on abaca fiber, CCF, and PP obtained by extrusion and injection molding [34]. The results showed that CCF generates better mechanical properties in biocomposites than abaca fiber. Tensile tests show that chemically modified CCF increases tensile strength up to 10% and tensile modulus up to 250% compared to PP. Finally, the authors conclude that based on fiber loading, biocomposites with 30% wt of fibers had the best set of mechanical properties among the materials studied. In another study, Perez-Fonseca et al. report the effect of the hybridization of CCF with agave fiber (up to 30%) and the addition of a coupling agent (maleated polyethylene, MAPE) on the water absorption and mechanical properties on HDPE based biocomposites obtained by extrusion and injection molding. Their results show that fibers and MAPE combination generates biocomposites with enhanced tensile and flexural strengths while lowering water absorption of the biocomposites. 

The reviewed literature presents an overview of the characterization of biocomposites based on chemically modified CCF [17,35]. However, less effort has been focused on the study and production of biocomposites based on untreated CCF and polyolefin blends using high-volume manufacturing processes such as extrusion and injection molding, which could be an advantage for the development of these materials in conventional plastic processing companies. In the present study, biocomposites based on a PP-HDPE blend and untreated CCF were prepared using extrusion following by injection molding. The thermal, mechanical, viscoelastic and morphological properties of the obtained materials were studied in order to evaluate CCF addition effect on the PP-HDPE blend behavior. Also, the analysis of the shrinkage and the dimensional stability of the injected specimens were studied through a novel three-dimensional microscopy study. 

## 2. Materials and Methods

### 2.1. Materials

**Figure 1:**
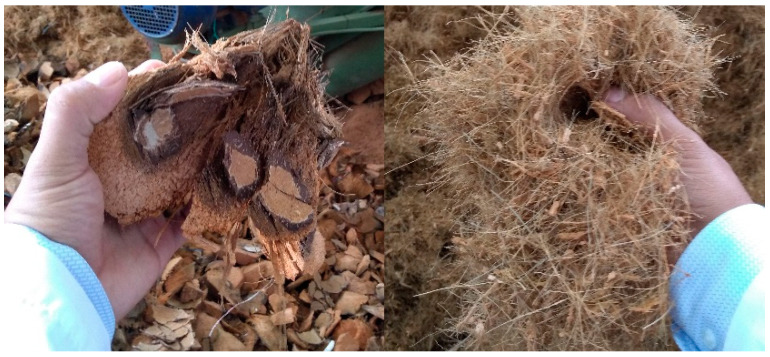
Raw coir coconut.

The polymeric materials used were an injection grade PP reference 575P and an injection grade HDPE reference M80064s with melt flow indexes (MFI) of 4.8 and 8.8 g/10 min respectively (measured at 190 °C, 2.16 kg). Both polymeric materials were purchased from SABIC (Al-Jubail, Saudi Arabia). The raw CCF, shown in Figure 1, were kindly supplied by “Super de Alimentos” (Manizales, Colombia) and were generated in the coconut candies production process. Before PP-HDPE-CCF biocomposites formulation, the CCF was grinded and sieved through a 400 μm sieve. 

### 2.2. Methodology

#### Neat Polymers and Biocomposites Processing

The extrusion process of the different materials was performed in a co-rotating twin-screw extruder with a L:D ratio 40:1 and a screw diameter of 22 mm, equipped with two volumetric feeders and a pelletizer located next to the die zone. During the extrusion, the following parameters were fixed:

- Neat PP, HDPE and a 50–50 (% *w*/*w*) PP-HDPE blend pellets were fed in the extruder feeding zone using a volumetric feeder
- For PP-HDPE-CCF biocomposites, the CCF was fed with another volumetric side feeder at L/D 20. The side feeder speed was variated to obtain PP-HDPE-CCF biocomposites with CCF loadings of 10%, 20%, and 30% (*w*/*w*)
- Temperature: 165 (feeding zone) to 185 °C (die)
- Twin-screw rotation speed: 50 rpm

Then the pellets of the different materials were dried in an X-DRY AIR mini dryer (Moretto, Massanzago, Italy) at 60 °C and a dew point of −52 °C. The specimens used for mechanical characterizations (described in Section 2.3.2) were injected in a microinjection molding machine BOY XS (BOY Machines Inc., Exton, PA, USA) with the following parameters:

- Temperature: 180 (feeding zone) and 185 °C (nozzle).
- Filling pressure: 60 to 80 bars
- Holding pressure: 60 bars
- Clamping force: 30 kN

**Figure 2:**
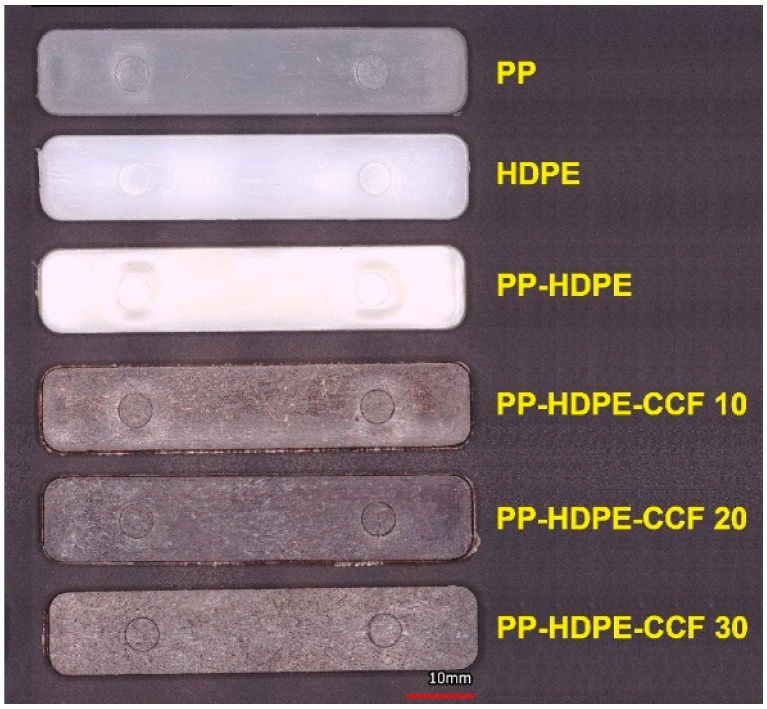
Injected specimens of neat PP, HDPE, PP-HDPE blend, and their biocomposites.

Figure 2 shows the injected flexural specimens of each material. The processing temperatures were set below 200 °C to avoid thermal degradation of the CCF (see Section 3.1), the remaining processing parameters were set based on reviewed literature regarding the processing optimization of natural fiber-polyolefin biocomposites [16,36]. 

The injection molding process allows to obtain complex geometric parts with fast function elements and in large quantities [37]. It offers several advantages over other manufacturing process as compression molding. According to Pickering [37], the content of natural fibers that can be incorporated during the injection process is between 20 to 40% by weight. A higher content is not recommended since an increase in fiber clearly reduces the flow capacity of the melt, generating instabilities during the process. Besides this, natural fibers have a similar morphology, but differ from each other by factors such as the internal area of the lumens, the number of lumens, the number and size of the fiber cells, the thickness of the secondary cell walls and the actual cross section [38]. These characteristics influence different fiber properties such as mechanical properties and bulk density which is related to the packing capacity of the fiber and the maximum throughput of the fibers [36]. In our case, the CCF throughput within the extruder was optimized to obtain PP-CCF biocomposites with a maximum fiber weight percentage of 30% and considerable flow properties to successfully apply in the injection molding process. 

### 2.3. Materials Characterization

#### 2.3.1. Melt Flow Index (MFI)

Melt flow index tests of neat PP and HDPE were performed at 190 °C and 2.16 kg using a plastics melt flow indexer. 

#### 2.3.2. Mechanical Properties

Tensile tests and three-point bending flexural tests were performed in a universal testing machine INSTRON Model 3366 (INSTRON, Norwood, MA, USA) equipped with an axial extensometer Epsilon model 3555 BP (Epsilon Tech, Jackson, WY, USA). Before being testing, the specimens were conditioned at 23 °C and 50% relative humidity for seven days. Tensile tests were performed on type V specimens (according to standard ASTM-D 638-14) at 23 °C, using a cross-head speed of 5 mm/min while flexural tests were carried out on bars with a rectangular cross-section at 23 °C, using a cross head speed of 1.3 mm/min, a distance between support spans of 50 mm and were performed up to 5% strain under standard ASTM D 790-17. The results were taken as the average of five samples. The impact strength of neat PP, HDPE, PP-HDPE blend, and their biocomposites was determined with an impact machine equipped with a 2.5 Joules pendulum. Notched IZOD impact tests were carried out at 23 °C and a starting angle of 150° under standard ASTM D 256-10 (2018). The results were taken as the average value of five samples. 

#### 2.3.3. Thermal Characterization

DSC tests were carried out using a TA Q2000 differential scanning calorimeter (Texas Instruments, Dallas, TX, USA) under nitrogen atmosphere at a scanning speed of 10 °C/min, with a sample of 10 mg in aluminum pans from 20 to 200 °C at a scanning speed of 10 °C/min, with a sample of 10 mg in aluminum pans. First, the samples were subjected to heating cycles at 10 °C/min from 20 to 200 °C to erase the thermal history related to processing events, following by cooling cycles at 10 °C/min from 200 to 0 °C. Finally, second heating cycles were performed at 10 °C/min from 0 to 200 °C. The samples were analyzed in aluminum crucibles under a N2 atmosphere. On the other hand, thermogravimetric analysis (TGA) tests were performed using a TA Q500 thermogravimeter (Texas Instruments, Dallas, TX, USA) from 25 to 600 °C at a heating rate of 10 °C/min. The samples were analyzed in aluminum crucibles under a N2 atmosphere. 

#### 2.3.4. Dynamic Mechanical Analysis (DMA)

DMA tests were performed using a DMA RSA-G2 (Texas Instruments, Dallas, TX, USA) in three-point bending mode from −80 to 150 °C, a frequency of 1 Hz, a constant heating rate of 3 °C/min and 0.01% of strain (taken from the linear viscoelastic domain of the plot E′ vs. strain reported earlier for PP and PE [31,32] and PP-natural fiber biocomposites [16,33]). Changes in storage modulus (E′), loss modulus (E″), and tan delta (loss factor) were recorded. 

#### 2.3.5. Morphology

Scanning electronic microscopy (SEM) of the PP, HDPE, PP-HDPE blend, and their biocomposites was performed using a Quanta FEG 250 microscope (ThermoFisher Scientific, Hillsboro, OR, USA) operating at a voltage of 10 kV. To obtain a brittle fracture on the visualized surfaces, the samples were immersed in a container with liquid nitrogen for 15 min. Later, the fracture was generated inside the container using two steel forceps. Finally, with the aim of increasing their electric conductivity, the samples were previously sputter-coated with gold. 

#### 2.3.6. Particle Size and Roughness Measurements

The particle size of the milled and sieved CCF was measured with a Three-dimensional microscope VR-3000 Series with a wide-area three-dimensional measurement system from Keyence (Keyence Corporation of America, Itasca, IL, USA). The roughness measurements were also performed twenty single milled fibers with this measurement system. The reported results were Ra (arithmetical mean height) and Rz (maximum height of profile). 

#### 2.3.7. Statistical Analysis

Tensile, flexural, and impact properties of the materials were subject to analyses of variance (ANOVA), and Tukey’s test was applied at the 0.05 level of significance. All statistical analyses were performed using Minitab Statistical Software Release 14 (Minitab LLC, State College, PA, USA). 

## 3. Results and Discussion

### 3.1. CCF Characterization

**Figure 3:**
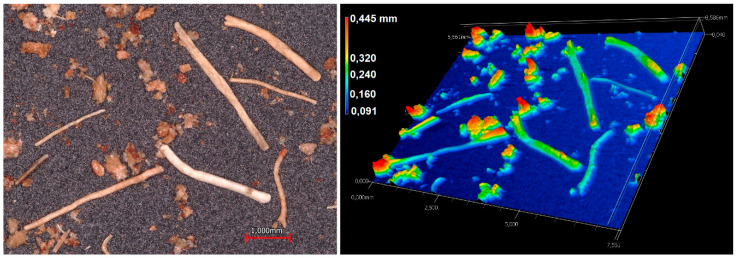
Optical micrograph and 3D height map of milled CCF.

**Figure 4:**
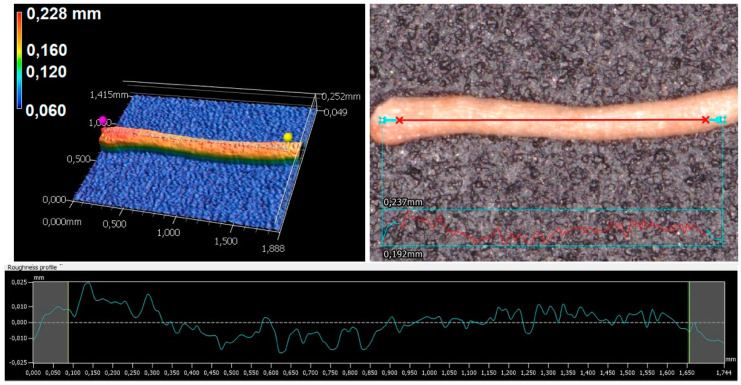
Roughness profile, optical micrograph and 3D height map of a single CCF.

The milled CCF (Figure 3) contains fibers and cork-like particles with an average length (l) of and 0.94 ± 0.22 mm and 0.38 ± 0.10 mm respectively. The average width (w) was 0.22 ± 0.04 mm for fibers and 0.29 ± 0.07 mm for particles. The average aspect ratio (*l*/*w*) of the milled CCF was 4.27 and 1.3 for fibers and particles. As shown in Figure 4, the wide-area three-dimensional measurement system allows to perform several measurements as the length, diameter and the roughness profile of a single natural fiber. Ra and Rz values obtained as the average value of twenty samples were 0.011 ± 0.004 mm and 0.055 ± 0.021 mm. The natural fibers surface roughness is an important parameter to measure because it plays a significant role in the mechanical interlocking between the fibers and matrix, which is related to biocomposites mechanical properties [18,26,39,40]. 

**Figure 5:**
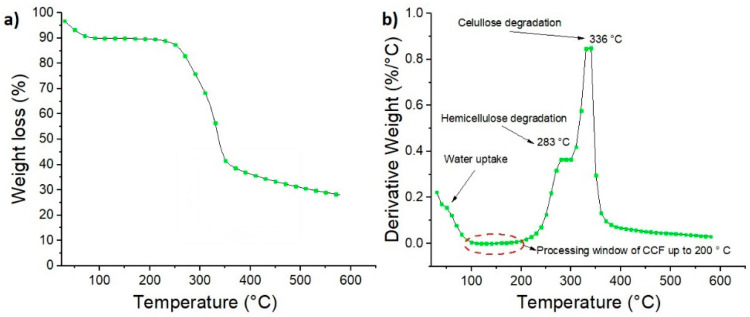
(**a**) TG and (**b**) DTG curves of coir coconut fibers at a heating rate of 10 °C/min.

Figure 5 shows the TGA and DTG thermograms of CCF under N_2_. Also, the degradation steps, onset temperature (To) and the maximum weight loss rate temperature of the sample (Tmax) are summarized in Table S1 supplementary information. For CCF, the TG curve shows four principal mass loss regions (Figure 5a). These regions are located around 30–100 °C, 100–200 °C, 200–300 °C, and 300–600 °C. The first region is related to the moisture evaporation of the sample with a weight loss of 9.2%. The second region is stable, without weight loss observed related to volatiles or CCF degradation by-products. This region shows that window processing of CCF based biocomposites should be below 200 °C (as indicates in the red circle in Figure 5b) to avoid thermal degradation of the fibers [41]. The third region between 200 °C and 300 °C presents a To of 253 °C and Tmax of 283 °C (Figure 5b), which is related to released elements from the sample like hemicellulose. The last region starts from 323 °C and with a Tmax 336 °C is related to α-cellulose decomposition. 

### 3.2. Biocomposites Characterization

#### 3.2.1. Mechanical Properties

**Figure 6:**
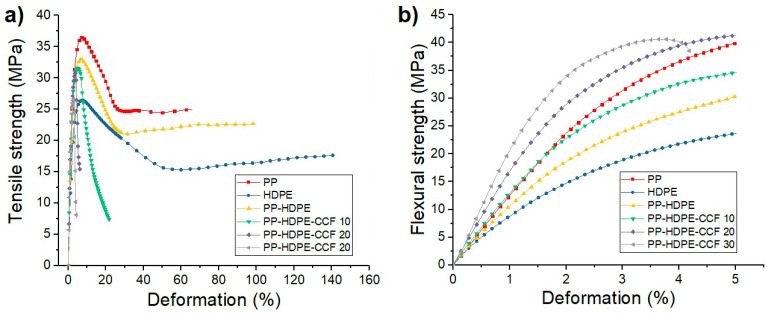
Average tensile stress vs deformation (**a**) and flexural stress vs deformation (**b**) of PP, HDPE, PP-HDPE blend, and their biocomposites.

The effect of CCF incorporation on the mechanical properties of the PP-HDPE blend was evaluated. The stress vs. strain graphs obtained from tensile and flexural tests for each material are shown in Figure 6. Also, the mechanical properties calculated from these tests are summarized in Supplementary Table S2. 

Regarding neat polymers, it is observed that PP tensile modulus (TM) and tensile strength (TS) values are higher than those of HDPE. However, HDPE deformation at break (εb) is higher compared to PP. For PP-HDPE binary blend, TM was not significantly different from PP (*p* ≥ 0.05) and its TS and εb values were between neat polymers values. In the case of biocomposites, the results of the test show that CCF addition generates significant increases (*p* < 0.05) in TM values of 16, 35, and 78% compared to the PP-HDPE blend for the biocomposites PP-HDPE-CCF 10, 20 and 30%. 

On the other hand, TS values of PP-HDPE- CCF 10, 20 and 30% decrease 6%, 7%, and 20%, respectively, in comparison with PP- HDPE blend (*p* < 0.05). Also, a CCF content increase generates a significant decrease in εb of the PP-HDPE matrix. This decrease in the TS and εb values have already been observed in some biocomposites [16,22,34] and could be related to the weak interfacial bonding between CCF (hydrophilic) and PP-HDPE (hydrophobic) or interface discontinuities that affect the biocomposites deformation capacity (see Section 3.2.4). With CCF loading increase, the weak interfacial area between the polymeric matrix and CCF increases, as a result, TS and εb values decreases. 

Flexural test results also show that biocomposites modulus values (FM) increase around 13 and 99% for biocomposites PP-CCF 10 and 30 respectively, compared with PP-HDPE. This stiffening effect is caused by a decrease in the polymeric chain mobility due to CCF and the higher mechanical properties of the CCF compared to the polymeric matrix and could become a decisive property in product applications where the rigidity (related to tensile and flexural modulus) is an essential factor. Also, the FS values of PP-HDPE and biocomposites presents significant differences (*p* < 0.05). FS values of the biocomposites increases between 14% and 35% for PPP-HDPE-CCF 10 and 30 respectively [42]. These differences observed in the strength values of both mechanical tests were also observed on PP-Rice Husk and PP-CCF biocomposites and can be due to a higher interaction natural fiber-polymeric matrix under compression stresses generates during bending [16,34]. 

The results of the Notched IZOD impact tests (Supplementary Table S2) shows that HDPE has a better impact performance than PP and could be related to its higher deformation and energy absorption capability. As expected, the PP-HDPE blend impact strength was 67% greater than that of neat PP (*p* < 0.05). On the other hand, CCF addition leading to a reduction of impact strength between 44 and 64% for PP-HDPE-CCF 10 and 30% respectively in comparison with PP-HDPE (*p* < 0.05), these results are in the range of those published by other authors for PP-CCF biocomposites [42,43]. This reduction in impact strength in the biocomposites with CCF load could be due to the stiffening effect of the matrix observed earlier in the tensile test and a weak interfacial adhesion between the CCF and polymeric matrix. Also, the increase of CCF generates fibers clusters within the biocomposites that could act as crack initiation sites [44]. 

#### 3.2.2. Thermal Properties

**Figure 7:**
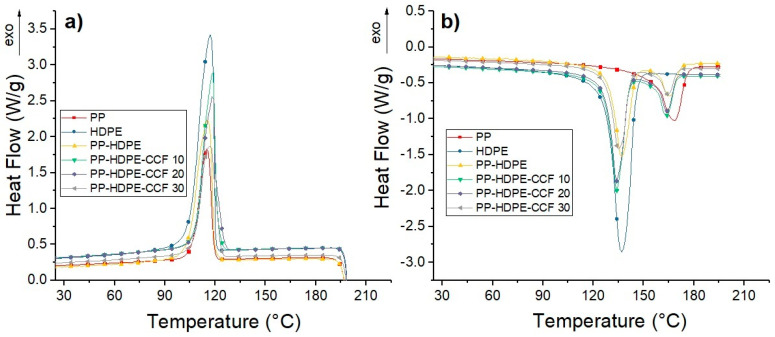
(**a**) Cooling and (**b**) Second heating DSC curves of PP, HDPE, PP-HDPE blend, and their biocomposites.

DSC thermograms for each material are shown in Figure 7. Also, the thermal properties obtained from these thermograms were included in Supplementary Table S3. The degree of crystallinity (*χ_c_*) of each material was estimated from Equation (1):

(1) $$\chi_{c}=\left(\frac{\Delta H_{m}}{\left[\Delta H_{m}^{0}*\left(1-W_{fiber}\right)*\left(w_{pol}\right)\right]}\right)*100$$

where *w_Fiber_* is the CCF fraction, *w_pol_* is the fraction of each polymer of the blend, Δ*H_m_* is the normalized melting enthalpy of each sample, and ∆*H*_*m*0_ is the specific melting enthalpy of 100% crystalline PP and HDPE. These values are 207 and 293 J/g, respectively [45]. 

The cooling cycle of PP, HDPE, and PP-HDPE blend (Figure 7a) shows an exothermic peak located between 115 and 117 °C, which corresponds to PP and HDPE chains crystallization. Regarding biocomposites, it is observed that during cooling CCF addition induces an increase of the PP-HDPE chains crystallization temperature around 2 to 4 °C. This increase indicates that CCF could act as a nucleating agent for polyolefin blends. 

The second heating curves (Figure 7b) show single melting endotherms located at 137 and 168 °C for neat PP and HDPE, respectively. These single peaks are associated with the melting temperature (Tm) of PP and HDPE crystals formed during the cooling stage. On the other hand, the PP-HDPE blend show two peaks located at 137 and 166 °C, related to the melting temperatures of each polymer when the blend is formed. This decrease has been observed in PP-PE blends and are related to a lack of miscibility between these polymers [9]. PP-HDPE-CCF biocomposites also exhibit two endothermic peaks around 135 and 165 °C related to the melting of the HDPE and PP phases. 

Considering Figure 7b, it is observed that there were small decreases in relation to the Tm of PP-HDPE-CCF biocomposites compared to neat PP-HDPE blend. This thermal behavior has been already observed in several studies regarding biocomposites as PLA-Ramie [46] and PP-NBr-Bagasse fibers [47]. This decrease (albeit small), is due to the incompatibility between non-polar hydrophobic matrices and polar hydrophilic untreated CCF fiber which leads to poor interfacial properties and thus lowering the melting point. Also, the presence of CCF could also disturb the chain arrangement in PP-HDPE blend, thereby decreasing the Tm of the corresponding biocomposites. 

**Figure 8:**
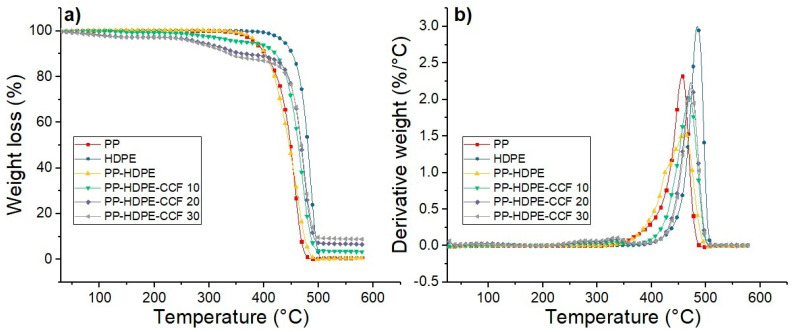
(**a**) TG and (**b**) DTG curves of PP, HDPE, PP-HDPE blend, and their biocomposites.

TG and DTG thermograms of each material are shown in Figure 8. Also, the thermal parameters obtained are summarized in Supplementary Table S1. 

PP and HDPE degradation occur in a one-step process with onset temperatures (To) located at 420 and 464 °C for PP and HDPE, respectively. Also, Tmax values were 457 °C for PP and 485 °C for HDPE. This result shows that HDPE has higher thermal stability than PP. PP-HDPE blend presents a single step degradation with a To located at 413 °C and two Tmax peaks located at 430 and 461 °C. These Tmax peaks are related to PP and HDPE phases of the blend. The residual char after 600 °C was 0.4 and 0.6% for neat polymers and PP-HDPE blend; this low residual char indicates that constituent atoms of the polyolefins (carbon and hydrogen) were volatilized during TGA test. 

On the other hand, the thermal degradation of biocomposites takes place in a multi-step process. The first step is related to CCF degradation and presents a To located between 266 and 275 °C, whereas the second step is associated with PP-HDPE thermal degradation. It is observed that CCF addition increases the polymeric matrix thermal stability, to values of PP-HDPE matrix increase between 28 and 40 °C and Tmax increases between 9 and 12 °C as compared to PP-HDPE blend. This increase of the polymeric matrix thermal stability given by CCF addition has already been observed in polyolefins-natural fibers biocomposites. This can be due to the increase of crystallinity and thermal properties of the matrix (as shown in Supplementary Table S3) generated by natural fibers nucleating effect [16,22]. Also, it is observed that the residual char of PP-HDPE-CCF biocomposites increases with the CCF content. 

#### 3.2.3. Dynamic Mechanical Analysis

**Figure 9:**
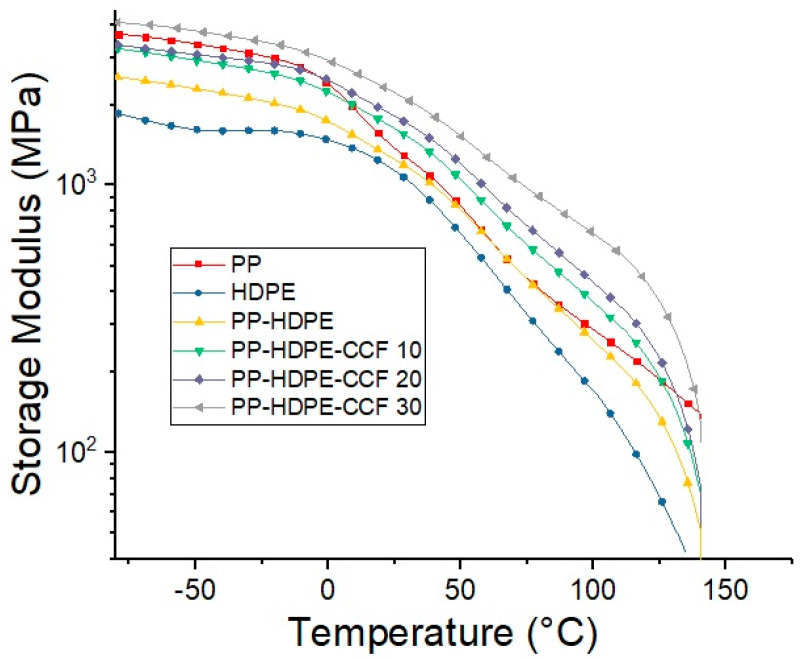
Temperature dependence of storage modulus of PP, HDPE, PP-HDPE blend, and their biocomposites.

Figure 9 and Table S4 presents the storage modulus values (E′) of each material vs. temperature. Regarding neat polymer matrices, E′ values of PP are higher than HDPE values in the entire temperature range, whereas, E′ of the PP- HDPE blend values were between neat polyolefins values. Also, E′ values decrease progressively with temperature increase for all materials. This could be due to the softening and the beginning of relaxation processes within the polymer matrix [48]. The CCF addition generates an increase of PP-HDPE blend stiffness, proportionally to the CCF content. This increase in E′ values are related to the stiffening effect given by rigid CCF and is consistent with the results obtained by tensile and flexural tests (Section 3.2.1). 

However, this stiffening effect is dependent on the glass transition temperature (Tg) of the PP phase. Below Tg (T = 0 °C), E′ increases 28% with 10% wt of CCF and shows a maximum increase of 65% with 30% wt of CCF compared to the PP-HDPE blend. At temperatures above Tg, for example T = 25 °C, this increase was between 31 and 78% for PP-HDPE-CCF 10 and 30% and is greater for temperatures above ambient (T = 80 °C) where the increase ranges from 35% to 125% with CCF content of 10% and 30% of respectively. This result suggests that at temperatures lower than Tg, the contribution of the fibers to the matrix rigidity is low since the matrix is in a glassy state. As the temperature increases, the drop in the matrix E′ values is compensated by the stiffness of the CCF fibers. In this case the E′ values is controlled by the percentage of fiber and increases with the fiber load in the biocomposite. 

**Figure 10:**
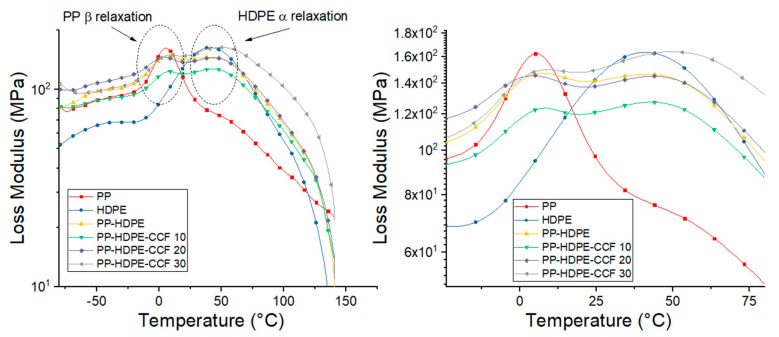
Temperature dependence of loss modulus of PP, HDPE, PP-HDPE blend, and their biocomposites.

On the other hand, loss modulus (E″) vs. temperature of HDPE, PP, PP-HDPE blend, and their biocomposites is shown in Figure 10. E″ plot of neat PP shows a β relaxation around 6 °C related to the glass transition temperature or Tg and a shoulder around 60 °C related to an α relaxation [49,50]. On the other hand, E″ plot of HDPE exhibit a broad α relaxation around 40 °C which is associated with the beginning of the molecular movement of the HDPE crystalline phase. The PP-HDPE blend reveal two peaks related to PP β relaxation and HDPE α relaxation which decline and shift towards high temperatures with 10% wt of CCF.These shifts to higher temperatures are caused by a decrease in the molecular movement of the PP and HDPE chains generated by the presence of the CCF and a dispersed phase on the matrix. With fiber loading increase, the E″ peaks intensity gradually increases and becomes broader. This behavior has been observed in polymer-natural fibers biocomposites [51,52] and reveal that CCF effectively suppress the polymeric chains mobility resulting in a broadening of the Tg range. 

**Figure 11:**
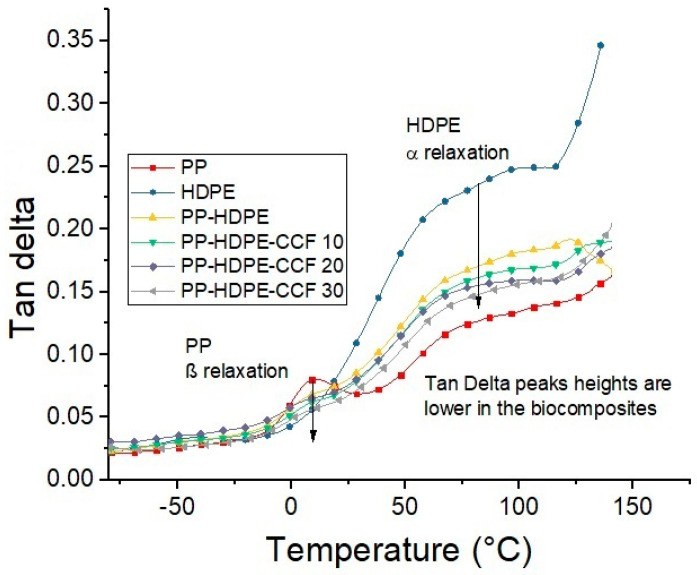
Temperature dependence of tan delta of neat PP, HDPE, PP-HDPE blend, and their biocomposites.

Figure 11 shows the variation of Tan δ with temperature. According to Saba et al. [51], Tan δ is the ratio between E″ and E′, and is related with the damping behavior of the polymeric matrix. This graph confirms that neat PP exhibit a β relaxation which corresponds to the glass transition (Tg) and a α relaxation between 60 °C to 75 °C [50], also neat HDPE present the α relaxation observed in E″ graphs (Figure 10). Regarding biocomposites, Tan δ peaks height related to the Tg of the PP phase and the α relaxation of the HDPE phase were observed to gradually decreases and shifts towards higher temperatures with CCF content increase (Supplementary Table S4).This result could be due to the amplified stiffness imparted by the CCF and confirms that fiber addition hinder the molecular movement related to the damping and could be an advantage in some applications where a better performance against mechanical loads is required. Also, the broadness of tan δ peaks, measured as the width at half maximum (FWHM), can be an indicator of the composite homogeneity and the interaction between the matrix and the fibers. In this sense, some author established that larger FWHM values implies heterogeneity and more contact between the phases of a biocomposite [46,53]. Table S4 shows that FWHM values of tan δ peaks is found to be the lowest for PP-HDPE blend and increases gradually with CCF content. This suggests that the heterogeneity of the biocomposite and the interaction between fibers and polymeric matrix increases with CCF content. 

**Figure 12:**
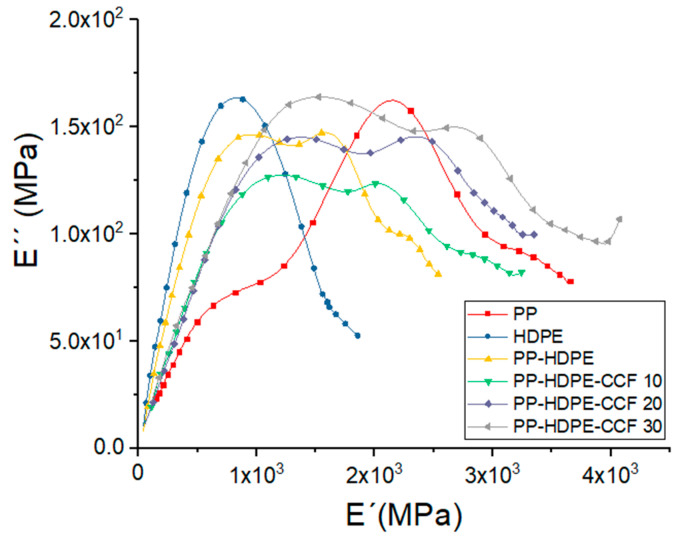
Cole-Cole plots of PP, HDPE, PP-HDPE blend, and their biocomposites.

With the aim to further understand the viscoelastic and structural behavior of the studied materials, Cole–Cole diagrams were evaluated. These diagrams are obtained by drawing E″ vs. E′, and can describe the nature of polymeric and composite materials [54,55]. A homogeneous material with a single relaxation time shows a semicircle diagram while a multiphase system with different relaxation times will show two irregularly shaped modified circles [54]. Figure 12 shows the Cole–Cole diagrams for PP, HDPE, PP-HDPE blend, and its biocomposites. The Cole–Cole curves shows that PP and HDPE are homogeneous systems with a concave shape (semicircles), while the PP-HDPE blend is a heterogeneous system which exhibit two semicircles due to two different relaxation mechanisms corresponding to the immisicibility between the polymeric phases. This result is in good agreement with the data obtained from the DSC test (Section 3.2.2). Regarding biocomposites, the Cole–Cole diagram also display two semicircles and a progressive increase in the values of the E′ and E″ with CCF loading [56]. This result show that CCF effectively suppresses the polymeric chains mobility and is an indicative of materials heterogeneity associated with greater differences in relaxation processes of the polymeric matrix when more CCF is incorporated. Also, the Cole–Cole diagram show that among the biocomposites, the one with 30% wt of CCF showed the highest E′ and E″ values. Therefore, it can be inferred that PP-HDPE-30 CCF biocomposite can absorb higher external loads while exhibiting elastic behavior maintaning its characteristics at higher temperatures [57]. 

#### 3.2.4. Morphology

**Figure 13:**
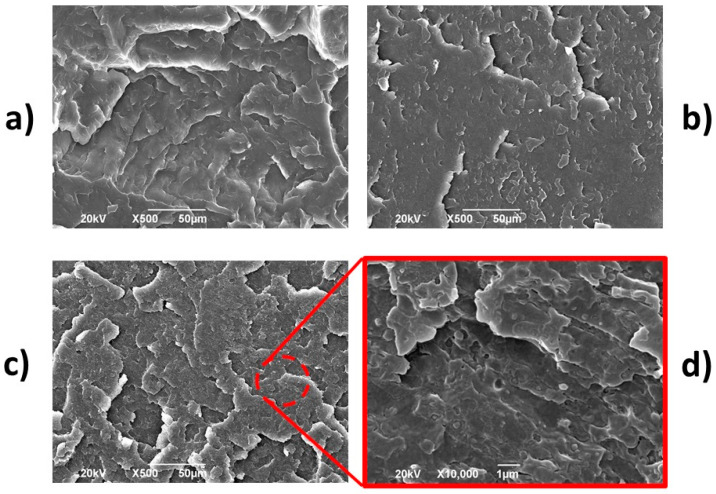
SEM micrographs for (**a**) PP, (**b**) HDPE and PP-HDPE blend (**c**,**d**).

SEM micrographs of neat polymers and PP-HDPE blend are shown in Figure 13a–c, respectively. For PP and HDPE, the fracture surface is smooth, and only one phase is observed in each sample. The PP-HDPE blend micrographs (Figure 13c,d) show a two-phase morphology due to PP and HDPE immiscibility [11]. This phase-separated morphology is consistent with the results obtained in DSC and DMA tests. According to the measured melt flow indexes (MFI values in Section 2.1), PP viscosity is higher in comparison with HDPE viscosity. Thus, PP is not efficiently sheared and separated during melt blending and is the dispersed phase of the blend. Also, HDPE with lower viscosity is the continuous phase [11]. The average diameter of the dispersed phase for the PP-HDPE blend was 0.32 ± 0.06 µm. 

**Figure 14:**
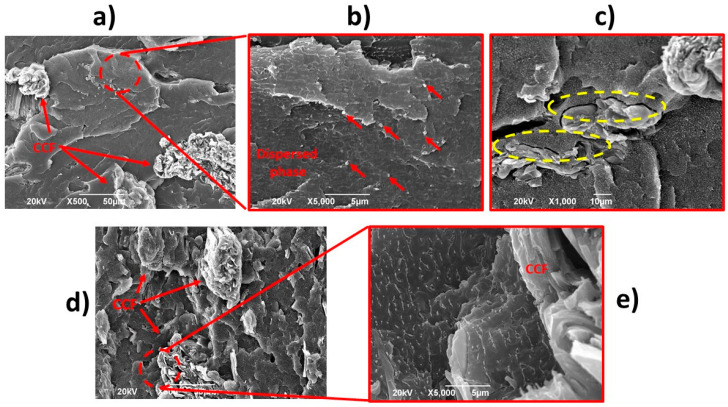
SEM micrographs for PP-HDPE-CCF 10 (**a**–**c**) and PP-HDPE-CCF 30 (**d**,**e**).

The biocomposite PP-HDPE-CCF 10 presents a rough fracture surface with a dispersed phase morphology (average diameter of 0.34 ± 0.08 µm) and dispersed CCF (Figure 14a,b). This result shows that 10% of CCF addition did not disturb the dispersed phase formation. Also, the interphase gaps between the CCF and the matrix (yellow circles in Figure 14c) indicate a poor interfacial adhesion between the polymeric matrix and CCF and could be related to the decreasing of tensile and impact properties (Section 3.2.1) [14,16,22]. On the other hand, for biocomposite PP-HDPE-CCF 30 (Figure 14d,e), the dispersed phase presents an oriented and elongated shape. In this case, a higher CCF proportion increases the contact surface between the polymeric phases and could reduce the surface tension between them. This behavior can change the spherical shape of the dispersed phase into the irregular spheroidal shape observed. 

#### 3.2.5. Linear Shrinkage and 3D Surface Characterization

**Figure 15:**
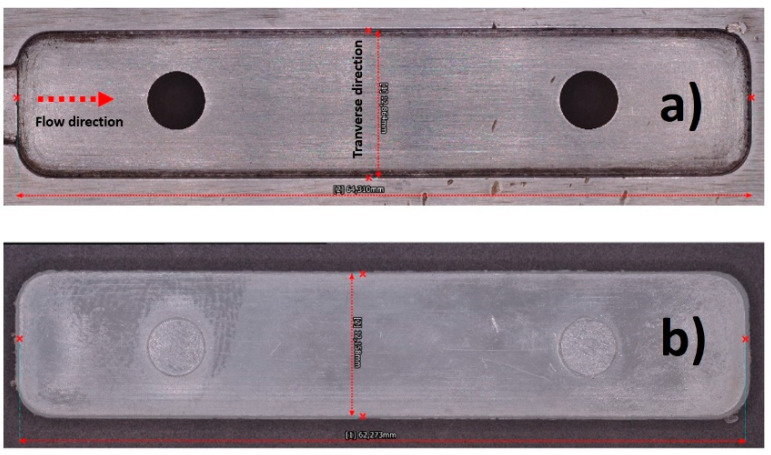
(**a**) mold cavity and (**b**) injected flexural specimen.

In this study, the shrinkage was determined as the difference among the linear magnitudes of the mold cavity and that of the injected specimen at room temperature two days after the injection [58]. Also, the following equations were applied to calculate the shrinkage of the molded specimens.

(2) $$Sf\left(\%\right)=\frac{L_{f\;\mathrm{specimen}}-L_{f\;\mathrm{mold}}}{L_{f\;\mathrm{mold}}}*100$$

(3) $$St\left(\%\right)=\frac{L_{t\;\mathrm{specimen}}-L_{t\;\mathrm{mold}}}{L_{t\;\mathrm{mold}}}*100$$

where *L*_f mold_ and *L*_t mold_ are the cavity mold dimensions, measured at the flow and transverse direction (as indicated in Figure 15a), *L*_f specimen_ and *L*_t specimen_ are the dimensions of the injected specimen in the two directions (Figure 15b). 

Linear shrinkage results of the specimens are shown in Table S5. These values are similar to those observed by Crawford et al., [59] for several polyolefins and show that all specimens present some degree of linear shrinkage during the injection process. However, it is observed that linear shrinkage decreases proportionally with the CCF content. Shrinkage is a frequent defect resulting from the injection molding process that can affect the quality and functionality of the final product. Some studies reported that overall shrinkage is affected by several parameters as the thermodynamic behavior of the injected polymer, the geometry of the injected part, the mold design, and the processing parameters among others [58,60]. 

**Figure 16:**
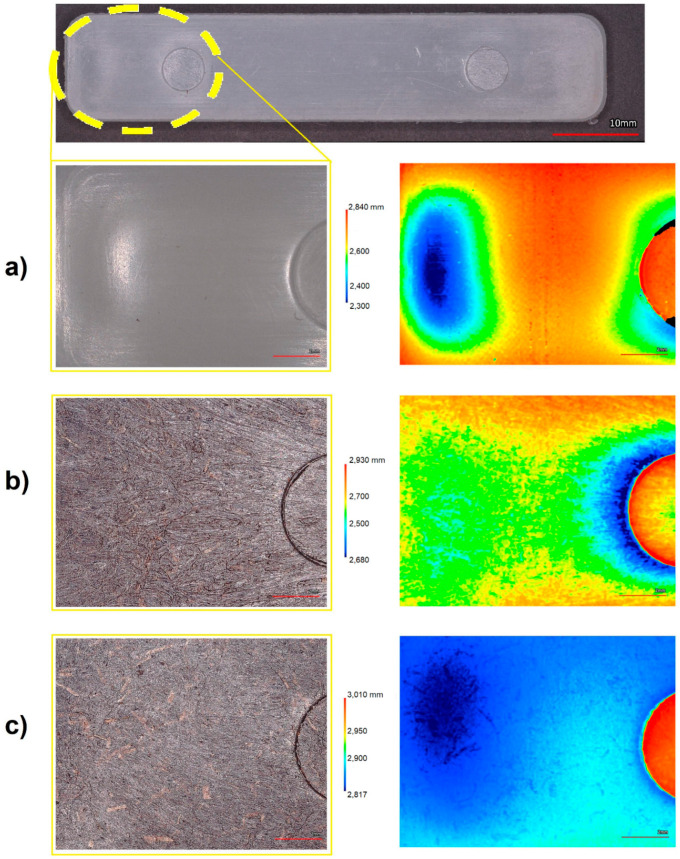
Surface characterization of (**a**) PP-HDPE blend (**b**) PP-HDPE-CCF 10 and (**c**) PP-HDPE-CCF 30 flexural specimens.

On the other hand, the surface characterization performed on the PP-HDPE blend and PP-HDPE-CCF 10, PP-HDPE-CCF 30 flexural specimens is shown in Figure 16. These measures were taken from the edge to the center of the specimen (as indicates in the yellow circle in Figure 16). For the specimen PP-HDPE (Figure 16a), the three-dimensional heights map shows a progressively decrease of the thickness values next to the edge of the specimen (blue and green zones) with a lower height value located at 0.541 mm. This effect is related to the shrinkage of the PP- HDPE matrix during the injection molding. Regarding biocomposites, a decrease in the shrinking with CCF addition was observed. For PP-HDPE-CCF 10 (Figure 16b) the lower height value was 0.250 mm whereas PP-HDPE-CCF 30 specimens present a lower height value located at the edge of 0.193 mm, which is uniform over the studied surface of the specimen (Figure 16c). 

This result shows that CCF particles addition enhance the dimensional stability of the PP-HDPE matrix and decrease manufacturing defects as shrinkage in injected specimens and could be an alternative for other additives commonly used to reduce injection molding defects in polyolefins such as talc, calcium carbonate or foaming agents [61]. Also, this behavior is in good agreement with those obtained by several researchers which studied the injection molding of biocomposites with engineering simulation and 3D design software and concluded that natural fibers addition reduces the appearance of processing defects as volumetric shrinkage and warpage in injection molding products [62,63,64]. 

## 4. Conclusions

In this research, PP-HDPE-CCF biocomposites (up to 30% CCF by weight) were processed using extrusion following by injection molding. The objective was to valorize the CCF for their use in polyolefin-natural fiber biocomposites. This CCF is an agro-industrial by-product of the Colombian food industry generated after the separation process of the coconut pulp. The characterization results show that CCF addition generates mechanical properties and thermal stability improvements without affecting the PP-HDPE melting behavior. Also, the dynamic mechanical analysis combined with the three-dimensional microscopy study analysis were sensitive tools for data generation that defines the dynamic mechanical properties, service temperatures and dimensional stability of polymers and biocomposites that support product development, particularly in construction and automotive applications. This study shows that CCF could be an alternative for other additives used to reduce injection molding defects such as talc, calcium carbonate or foaming agents. Finally, PP-HDPE-CCF biocomposites are alternative materials for the design and manufacture of products by injection molding that due to their availability and recyclability potential could generate some economic and environmental benefits in the search for sustainability in the plastics industry facing a circular plastics economy. 

## Supplementary Materials

The following are available online at https://www.mdpi.com/2073-4360/12/7/1507/s1, Table S1: Thermogravimetric data of the studied materials, Table S2: Mechanical properties of the studied materials, Table S3: Differential scanning calorimetry data of the studied materials, Table S4: DMA results of the studied materials, Table S5: Linear shrinkage of injected specimens at flow (Sf) and transverse (St) directions.
