# Supplementary material for: Injection Molding of Coir Coconut Fiber Reinforced Polyolefin Blends: Mechanical, Viscoelastic, Thermal Behavior and Three-Dimensional Microscopy Study
Source: Polymers (Basel). 2020 Jul 7;12(7):1507. doi: 10.3390/polym12071507 (PMC7408383; doi:10.3390/polym12071507)
Supplement: Supplementary file 1 [file polymers-12-01507-s001.pdf]

Table S1. Thermogravimetric data of the studied materials.

Sample	Degradation step	T_{0nset} (°C)	T_{max}(°C)	Residual Char (%)
CCF	1	253	283	28
	2	323	336	
PP	1	420	457	0.4
HDPE	1	464	485	0.4
PP-HDPE	1	413	430-461	0.6
PP-HDPE-CCF 10	1	266	334	3.3
	2	441	470	
PP-HDPE-CCF 20	1	273	337	6.8
	2	452	472	
PP-HDPE-CCF 30	1	275	337	8.9
	2	453	473	

Table S2. Mechanical properties of the studied materials.

Material	Mechanical Properties*					
	Tensile properties			Flexural properties		Impact properties
	Modulus (MPa)	Strength (MPa)	Deformation at break (%)	Modulus (MPa)	Strength (MPa)	Impact Strength (J/m)
PP	1700 ± 109[a]	36.7 ± 0.5[a]	65.4 ± 5.2[a]	1278 ± 45[a]	39.9 ± 0.8[a]	23.1±1.9[a]
HDPE	1360 ± 85[b]	26.0 ± 1.0[b]	141.1 ± 0.5[b]	1052 ± 39[b]	23.7 ± 0.3[b]	53.0±2.6[b]
PP-HDPE	1656 ± 58[a]	33.5 ± 0.8[c]	99.5 ± 5.3[c]	1159 ± 31[c]	30.3 ± 0.6[c]	38.8±2.6[c]
PP-HDPE -CCF 10	1934 ± 81[c]	31.3 ± 0.7[d]	31.3 ± 22.4[d]	1308 ± 42[d]	34.6 ± 0.3[d]	21.5±2.7[a]
PP-HDPE -CCF 20	2232 ± 156[d]	30.9 ± 0.7[d]	6.1 ± 0.9[e]	1804 ± 108[e]	41.3 ± 2.2[e]	14.6±1.1[d]
PP-HDPE -CCF 30	2962± 146[e]	26.9 ± 0.9[b]	3.9 ± 0.2[f]	2309 ± 246[f]	40.9 ± 1.8[e]	13.9±0.5[d]

a–f Different letters in the same column indicate significative differences ($p < 0.05$).

*Mean of five replications ± standard deviation

Table S3. Differential scanning calorimetry data of the studied materials.

Sample	Cooling	Second heating					
		HDPE phase			PP phase		
	Tc ºC	Tm ºC	ΔHm (J/g)	Xc (%)	Tm ºC	ΔHm (J/g)	Xc (%)
PP	115	-	-	-	168	87.5	42
HDPE	117	137	195	70	-	-	-
PP-HDPE	115	137	82.2	59	166	21.9	21
PP-HDPE-CCF 10	119	134	93.7	75	164	25.9	28
PP-HDPE-CCF 20	119	134	88.6	80	165	22.3	27
PP-HDPE-CCF 30	117	135	74.3	76	165	16.4	23

Table S4. DMA results of the studied materials

Sample	E´ (MPa)			Tg (ºC)*	α relaxation (ºC)*	Full width at half maximum (FWHM) of tan δ peaks**		tan δ peaks height	
	-50ºC	25ºC	80ºC	PP Phase	HDPE phase	PP Phase	HDPE Phase	PP Phase	HDPE Phase
PP	3389	1382	407	8.9	-	22.2	-	0.08	-
HDPE	1625	1136	287	-	58.6	-	63.3	-	0.22
PP-HDPE	2289	1250	401	5.1	66.3	14.4	53.8	0.07	0.17
PP-HDPE-CCF 10	2936	1632	542	6.7	68.0	16.5	54.1	0.06	0.16
PP-HDPE-CCF 20	3086	1822	641	5.5	69.0	20.4	54.9	0.06	0.14
PP-HDPE-CCF 30	3791	2225	902	6.9	71.6	23.9	56.3	0.05	0.12

*Relaxation values were taken at the maximum peak of tan delta curves.

** FWHM values were taken after a baseline correction of tan delta curves.

Table S5. Linear shrinkage of injected specimens at flow (Sf) and transverse (St) directions

**Sample**	**Linear Shrinkage**	
	**Sf (%)**	**St (%)**
PP	2.5	5.7
HDPE	3.7	6.4
PP-HDPE	3.0	6.3
PP-HDPE-CCF 10	2.5	5.7
PP-HDPE-CCF 20	2.3	5.2
PP-HDPE-CCF 30	1.9	4.6
